# Comparison of Machine Learning Algorithms For Beck Depression Inventory Measured Depression Status Classification

**DOI:** 10.1192/j.eurpsy.2023.902

**Published:** 2023-07-19

**Authors:** M. Ballı, A. Okan, N. Ö. Gürsan, S. Gülgöz, H. Yapici Eser

**Affiliations:** 1 Neuroscience, Koc University, İstanbul, Türkiye; 2 Psychology, University of North Carolina at Chapel Hill, Chapel Hill, United States; 3 Computer Engineering; 4 Psychology; 5 School of Medicine, Koc University, İstanbul, Türkiye

## Abstract

**Introduction:**

Depression is a psychiatric disorder characterized by low mood and anhedonia. The diagnosis of depression and the initiation of treatment is important for improving quality of life and avoiding disability. Machine learning (ML), can be used for solving classification and regression problems.In this study, scores of multiple psychiatric scales were used to detect depression and different ML algorithms were used to study if they can help for diagnosing depression accurately.

**Objectives:**

The purpose of the study is to detect with high accuracy whether people are depressed or not by using widely used ML algorithms. It is also aimed to compare the algorithms used to predict depression with each other.

**Methods:**

Data were collected from 96 university students. Beck Depression Inventory (BDI), Beck Anxiety Inventory, Neo Personality Inventory, Chronic Stress Scale (CSS), Perceived Stress Scale (PSS), Childhood Trauma Questionnaire, Post-Traumatic Stress Disorder Checklist (PTSD), SHAPS, Relationship Scales Questionnaire and Dissociative Events Scale were applied. 14 points from the BDI was accepted as the cut-off value as depressed. Total scores of each scale was used as the dependent variable in the Xgboost (XGB) to classify the depression. By XGB, the most important 4 of these surveys and scales were selected to use in the Non-Linear (NL) models such as XGB, Decision Trees (DT), Support Vector Machines (SVM), K-Nearest Neighbor (KNN). Lastly, a linear model as a Logistic Regression (LR) model was also used to compare with the NL algorithms. The success of the models was measured with the Cross Validation method, which is the gold standard in ML.

**Results:**

In the model in which all measurements are used as Independent Variables (IV), the XGB highlighted 4 scale scores: these are CSS, PSS, SHAPS and PTSD. All scale scores were used as IV, both XGB and DT classified depression with a success of 87.5%, while this score increased to 89.6% in both models when 4 prominent scales’ scores were used as IV. In the KNN, the classification made with prominent scales increased the success from 83% to 86%. The variance explanation rate of the LR model using 4 prominent scales remained at 58%.

**Conclusions:**

With ML’s ability to solve NL relationships and dimensional reduction ability, models in which a large number of variables are input and there is no high correlation between dependent variables and IV can be classified with high success. Also, the success of the models was increased by choosing the most importants of the many IV and the variables that contributed negatively to the model could be excluded. The use of ML can yield promising results in fields such as psychiatry where linear relationships cannot be observed much.

**Disclosure of Interest:**

None Declared

